# Suppression of *Aedes albopictus* in Sri Lanka using the Sterile Insect Technique (SIT) with a sustained effect

**DOI:** 10.1051/parasite/2025050

**Published:** 2025-09-17

**Authors:** Menaka Hapugoda, Nilmini Silva Gunawardene, Tharaka Ranathunge, Sudath Samaraweera, K. Karunathilake, Bazoumana B.D. Sow, Gayan Parakrama Withanage, Indika Weerasinghe, Hamidou Maiga, Jeremy Bouyer

**Affiliations:** 1 Molecular Medicine Unit, Faculty of Medicine, University of Kelaniya Ragama Sri Lanka; 2 Department of Zoology, Faculty of Science, Eastern University Batticaloa Sri Lanka; 3 National Dengue Control Unit Public Health Complex 555/5, Elvitigala Mawatha Narahenpita Colombo 05 Sri Lanka; 4 Department of Sociology, Faculty of Social Sciences, University of Kelaniya Kelaniya Sri Lanka; 5 Institut de Recherche en Sciences de la Santé (IRSS) Bobo-Dioulasso 01 BP 545 Burkina Faso; 6 Insect Pest Control Subprogramme, Joint FAO/IAEA Centre of Nuclear Techniques in Food and Agriculture, Department of Nuclear Sciences and Applications, International Atomic Energy Agency A-1400 Vienna Austria; 7 UMR ASTRE (Animal Santé Territoires Risques et Ecosystèmes), CIRAD, Plateforme CYROI 2 rue Maxime Rivière 97491 Sainte-Clotilde La Réunion France

**Keywords:** Sterile male mosquitoes, Suppression, Integrated Vector Management, Vector control, Irradiation, Dengue

## Abstract

Dengue fever remains a significant public health concern in Sri Lanka, leading to recurrent epidemics and imposing substantial socio-economic burdens. This study aimed to assess the efficacy of the Sterile Insect Technique (SIT) against *Aedes albopictus* (Skuse), the predominant dengue vector in the country, through a pilot field trial of an Integrated Vector Management (IVM) strategy including the SIT. The pilot trial was conducted in the Gampaha district, which reports the second-highest number of dengue cases in the country. A total of 3,300,000 sterile males, exposed to a 50 Gy radiation dose, were released over 33 weeks (100,000/week) within a 30-hectare release area. Entomological assessments were conducted at 115 trapping stations over a period of 71 weeks (October 2020–August 2022). Induced sterility of 98.16% in mosquito eggs was reached within the release area as compared to the control area (binomial generalized linear mixed model, deviance 2.408, df = 2, *p* = 0.016), indicating a notable impact of the SIT. The trial achieved nearly 98% suppression of adult vector mosquitoes, with a sustained suppression effect for 13 weeks post cessation of releases. These findings suggest that SIT can be effectively integrated as a potential additional tool into the future IVM strategy in Sri Lanka.

## Abstract (fr)

La dengue demeure un problème de santé publique majeur au Sri Lanka, entraînant des épidémies récurrentes et imposant un lourd fardeau socio-économique. Cette étude visait à évaluer l’efficacité de la technique de l’insecte stérile (TIS) contre *Aedes albopictus* (Skuse), le principal vecteur de la dengue dans le pays, grâce à un essai pilote sur le terrain dans le cadre d’une stratégie de gestion intégrée des vecteurs incluant la TIS. Cet essai pilote a été mené dans le district de Gampaha, qui enregistre le deuxième plus grand nombre de cas de dengue du pays. Au total, 3 300 000 mâles stériles, exposés à une dose d'irradiation de 50 Gy, ont été relâchés pendant 33 semaines (100 000/semaine) dans une zone de 30 hectares. Des suivis entomologiques ont été menés dans 115 stations de piégeage sur une période de 71 semaines (octobre 2020-août 2022). Une stérilité induite de 98,16 % des œufs de moustiques a été atteinte dans la zone de lâcher par rapport à la zone témoin (modèle mixte linéaire généralisé binomial, écart-type 2,408, ddl = 2, *p* = 0,016), indiquant un impact notable de la TIS. L’essai a permis d’obtenir une suppression de près de 98 % des moustiques vecteurs adultes, avec un effet de suppression persistant pendant 13 semaines après l’arrêt des lâchers. Ces résultats suggèrent que la TIS peut être efficacement intégrée comme un outil supplémentaire potentiel dans la future stratégie de contrôle intégré des vecteurs au Sri Lanka.

## Introduction

Dengue is a major mosquito-borne disease prevalent in tropical and subtropical regions, caused by the Dengue Virus (DENV). Transmission of dengue is mainly influenced by large-scale unplanned urbanization and increases in human population density. “Dengue” is responsible for more illness and deaths than any other arboviral diseases of humans [26]. About half of the world’s population is now at risk of dengue with an estimated 100–400 million infections occurring each year [46]. Almost all countries in the South-East Asia region are dengue endemic. Five of them, including Sri Lanka, are among the 30 countries with the highest burden of dengue in the world [47].

Dengue Fever (DF)/Dengue Hemorrhagic Fever (DHF) is endemic throughout Sri Lanka, with frequent and cyclical epidemics. From the year 2000 to 2020, mass dengue epidemics occurred in Sri Lanka in the years 2004, 2009, 2012, 2017, and 2019 and the largest epidemic was reported in 2017 (866 per 100,000 population; 186,101 cases), followed by 2019 (479.7 per 100,000 population; 105,049 cases) [1]. There was also a significant increase in dengue cases in 2023 in Sri Lanka, accompanied by an atypical epidemiological trend [2].

This disease has a significant effect on the livelihoods of people living in the endemic areas of the country. Most dengue patients are reported from the Western Province of Sri Lanka every year. The second highest number of dengue cases has been reported in the Gampaha district of the Western Province since 2010 [48].

Dengue control efforts have been targeted at the disease and vector, including dengue infections in patients, clinical management of DF/DHF patients, Integrated Vector Management (IVM) and social mobilization [35]. Further, emergency response during outbreaks in terms of accelerated vector control and public awareness through the media is conducted. The government spends a considerable amount on controlling dengue in the country [43], and at the same time, infected people lose their working hours and income.

Dengue vector control has become the most important strategy in control of the disease in the country. Strengthening vector control measures through the integration of innovative strategies is essential [28]. Application of innovative dengue vector control methods is very important since conventional techniques currently available are neither adequate nor sustainable for IVM. The Sterile Insect Technology (SIT) [29] has been used for more than 60 years for agricultural pest management and is now being increasingly applied to mosquitoes as part of IVM programs [30]. This technique is emerging as a powerful complement to most commonly used approaches, in part because this technique is environmentally friendly, species-specific, and non-persistent in the environment if releases are stopped [27, 37]. Sterile male mosquitoes are released into target environments, where they mate with wild females, resulting in infertile eggs.

This process may result in suppression of the vector population and the reduction of dengue cases in the selected area. The SIT has seen significant developments against *Aedes* mosquitoes.

The International Atomic Energy Agency (IAEA) and World Health Organization (WHO) have developed a framework for SIT named Phased-Conditional Approach (PCA) [45]. SIT needs to be implemented using the PCA which contains different phases, and the implementation of the next phase is conditional upon completion of activities in the previous phase [9, 45]. The phases are namely i) baseline data collection, ii) small scale field trials, iii) pre-operational program, and iv) operational program. Recent progress in the development of this SIT package against mosquitoes has made it possible to consider its larger scale deployment [45]. There are 39 globally distributed sterile male releasing projects, including 34 in Phases-I and II, 4 in Phase-III and 1 in Phase-IV as of 2024 [11].

Laboratory and semi-field evaluations of the SIT for *Aedes albopictus* (Skuse), the most widespread dengue vector in the country were completed in Sri Lanka, during Phase 1 [22, 23]. Additionally, during Phase I, a series of Mark-Release-Recapture (MRR) experiments were conducted to evaluate the field performance of sterile male mosquitoes and estimate wild population sizes for future SIT trials [21]. The overall objective of this study was to test the impact of the SIT on *Ae. albopictus* within a pilot field trial (Phase II) and to measure persistence of the suppression after stopping releases of sterile males.

## Materials and methods

A case control trial covering two areas with similar geographical, environmental, and socio-economic characteristics was conducted as an SIT pilot field trial (Phase II) in Sri Lanka.

### Ethical and regulatory aspects

The study was approved for ethics by the Ethics Committee, Institute of Biology, Sri Lanka (ERC IOBSL 207.02.2020). Releasing mosquitoes was permitted by the National Regulatory body, Biodiversity Secretariat, Ministry of Mahaweli Development, Sri Lanka. Agreement for male mosquito releases was obtained by the Education, Training and Research Unit, Ministry of Health, Sri Lanka. All methods were performed in accordance with the relevant guidelines and regulations [34]. Informed written consent was obtained from households to place mosquito traps at their premises and conduct entomological surveillance.

### Establishment and maintenance of an *Ae. albopictus* colony

A local strain of *Ae. albopictus* was colonized using wild specimens collected from the release area. The mosquito colony was maintained in cages (24 cm × 24 cm × 24 cm BugDorm; MegaView Science Co., Ltd, Taichung, Taiwan) at a density of 1,000 individuals per cage with mesh screening on top, under a 12:12 h (light:dark) cycle at standard conditions (27 ± 2 °C and 75 ± 5% humidity) in a confined insectary at the Molecular Medicine Unit, Faculty of Medicine, University of Kelaniya, Sri Lanka. Cages were supplied with 10% sugar solution every other day starting from the first day of emergence. A blood meal of cattle origin was given after the 3^rd^ day of emergence, and a second blood meal was given one week after the first using a Hemotek (PS-6 System, Discovery Workshops, Accrington, UK). After blood feeding, sugar cups were placed inside the adult cages for feeding. Egg laying cups with a strip of white Whatman filter paper (27 cm long and width 5 cm) and filled with dechlorinated water (100 mL) were placed inside the cage after 48 h of blood feeding. Two egg papers with approximately 10,000 eggs were collected from a single generation in the cage. Egg papers were removed from the cage and air dried and kept into a plastic container at room temperature until used. Egg papers (10,000) were transferred into a glass bottle with lukewarm water (1 L) and kept overnight. On the following day, hatched eggs were transferred into a white plastic larval raring tray (30 cm × 40 cm × 8 cm) with 1,000 mL dechlorinated water and maintained at the density of 1 larva/1 mL, 1,000 larvae per tray.

Larval diet (21.5 g tuna powder, 3.5 g % Brewer’s yeast in 100 mL of water) was provided according to the following regime; 5 mL the first 3 days (daily twice-morning and evening) and the amount later increased to 8–10 mL, depending on the size of the larvae for the 4^th^ and 5^th^ days [42]. Pupae (500) were transferred into plastic containers (5 cm radius) with 150 mL water and kept inside a cage until emergence of adults.

### Sex separation

Male *Ae. albopictus* pupae (24–48 h of age) were separated based on size using a Fay-Morlan glass plate sorter (M5412, John W. Hock Company, Gainesville, FL, USA) [12, 14].

### Irradiation

Male pupae (1,000) aged 48 h were transferred into pupal cups with water (500 mL) and transported to the irradiation facility at 25 °C. Water level of the pupal cup was reduced to 50 mL and exposed to a pre-determined dose of 50 Gy dose of Co-60 for 2 min and 8 s [22, 39] using an irradiator (GammaCell 220, Atomic Energy of Canada Ltd., Chalk River, ON, Canada) located in the Horticultural Crop Research and Development Institute, Sri Lanka. After irradiation, water level was increased again up to 500 mL and pupae were brought back to the laboratory. Pupal bowls were placed into mosquito cages (24 cm × 24 cm × 24 cm) under standard laboratory conditions [26 ± 1 °C, 75–80% RH and a photoperiod of 12:12 h (L:D)] with 10% sucrose solution for 2 days until all pupae became adults.

### Transporting sterile male mosquitoes to the release area

Adult sterile male mosquitoes were transported weekly (100,000) to the release area in plastic cups (500/cup) at room temperature since the release area was situated within 17 km from the facility.

### Release and control areas

Kidagammulla Grama Niladhari (GN) division in the District of Gampaha (6°54′5″ and 7°20′), a geographically isolated semi-urban area of 30 ha surrounded by paddy fields and a wide road situated in the West part of Sri Lanka was selected as the release area. Yakkala South GN (79°4′75″ and 80°13′) located 1,260 km linearly apart from the release area with the same size was selected as the control area. The areas have similar geographical, environmental, and socio-economic characteristics. Both areas are residential, with approximately 570 landed houses in each. Entomological monitoring activities conducted in the past have confirmed that *Ae. albopictus* is the only established *Aedes* mosquito present in both the release and control areas, and the buffer zone [24].

### Mapping release and control areas

**Figure 1:**
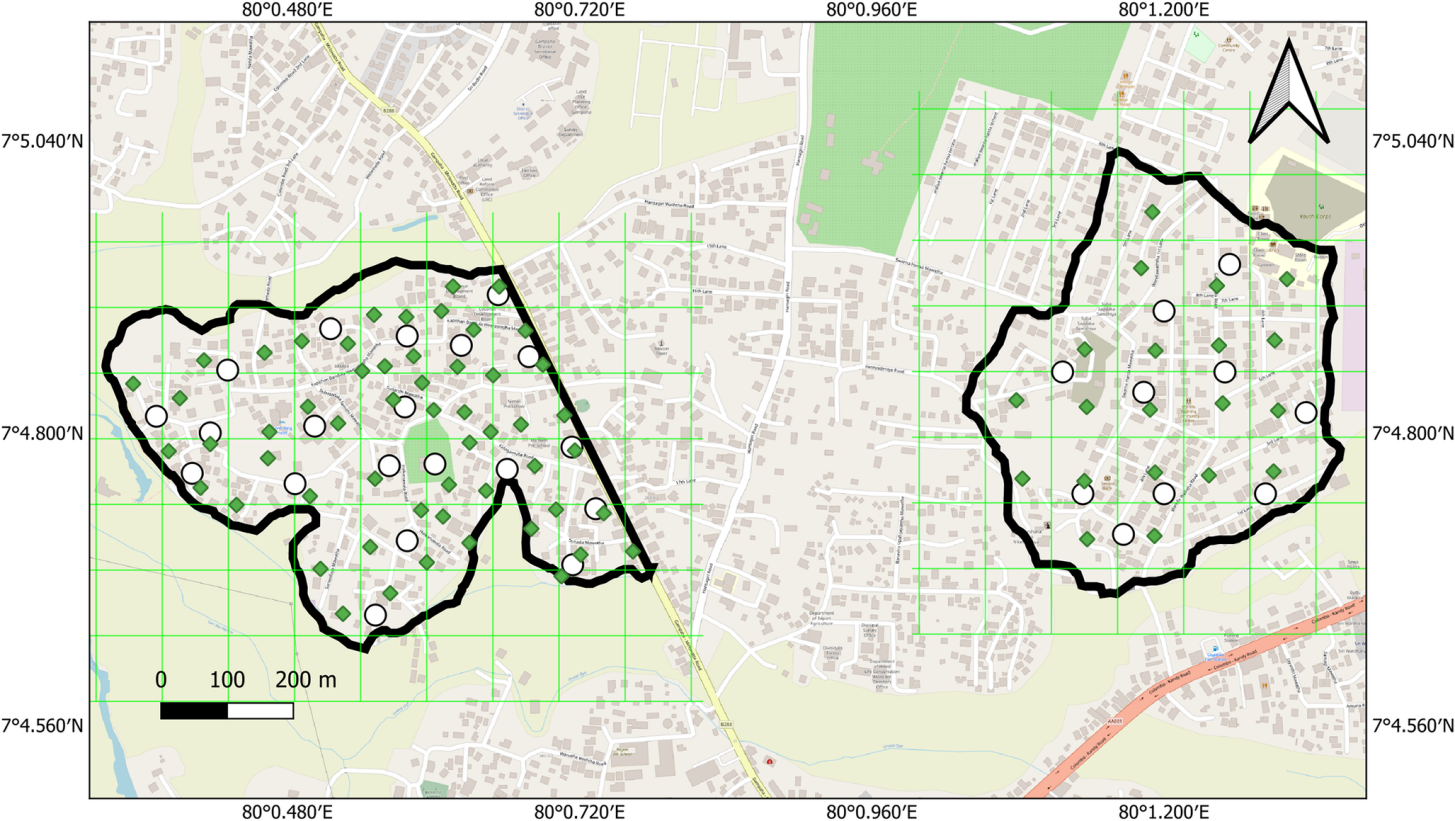
Map showing the release (Kidagammulla) and control (Yakkala South) areas in Gampaha District, Sri Lanka, with 100 m × 100 m grids and trap sites.

The satellite images of the selected release and control areas were studied to identify vegetation cover and house density. Geographical Information System (GIS) based maps were developed using QGIS, version LTR 3.34.6 for both areas using OpenStreetMap as basemap with the World Geodetic System (WGS) 84 (EPSG:4326) coordinate system. The location of each trapping point distributed equally in the release area [56 ovitraps and 20 Bio Gene (BG) sentinel traps] and control area (20 ovitraps and 10 BG traps) (Supplementary file S1) was incorporated into the map of each area. Only trapping stations for ovitraps (5) were used in the buffer zone to measure the emigration rate of sterile females. Identification numbers for release and trapping points were recorded in the database. Grids of 100 m × 100 m were prepared using the software and geographically overlaid on the GIS map, and the middle of each cell was selected as the release point (20) in the release area (Fig. 1).

### Stakeholder and community awareness

Intensive community and stakeholder awareness programs were conducted before mosquito releases, as this technique was a novel tool in Sri Lanka. A stakeholder and community awareness campaign highlighting the safety of the release of sterile male mosquitoes was conducted by a social mobilization team. In this effort, the team conducted Focus Group Discussions (FGDs) and community awareness meetings to convince the inhabitants, government officers, and community leaders of the area about the study [21]. Further, door-to-door visits, distributing leaflets, and introduction of an emergency contact point were also conducted under the program.

### Releasing sterile male mosquitoes in the release area

The release parameters, frequency, location, and release rate were set up based on the mosquitoes’ average life expectancy, flight range, and wild male abundance, respectively, as estimated by an MRR trial. Weekly mosquito releases at a density of more than 3,333 sterile males/ha at release points situated about 100 m apart from each other were conducted under the current study. We used the results of a previous MRR study in the treated area [21] to select the release density. This release density was set to approximately 20 times the mean wild male mosquito population density (163 males/ha), since the goal was to reach a mean ratio of sterile to wild males of 10 throughout the week with only one release per week, accounting for mean life expectancy of the sterile males of 3.55 ± 2.32 days after release. Mosquitoes were released early in the morning to avoid mortality and clumping. They were released immediately after transport to the field by placing open cups at 20 release points (5,000/per point) in a position exposed to the sun.

### Monitoring protocol

Entomological impact was monitored over a total period of 96 weeks (approximately 22 months), spanning from October 23, 2020 to August 26, 2022 including: Pre-intervention monitoring (October 23, 2020–January 15, 2021; 12 weeks) establishing baseline mosquito activity prior to interventions, intervention part 1 (January 15, 2021–May 14, 2021; 17 weeks) following on immediately and focusing on initial control measures, a breakdown period (May 14, 2021–June 25, 2021; 6 weeks) providing a temporary pause between interventions, intervention part 2 resuming on June 25, 2021 and continuing for 35 weeks until February 25, 2022, covering seasonal variations, and Post-intervention monitoring (February 25, 2022–August 26, 2022; 26 weeks) assessing the long-term effects after the intervention ceased. Monitoring throughout all phases utilized ovitraps (for egg surveillance) and BG traps (for adult mosquito capture), ensuring continuous data collection on *Aedes* species dynamics and intervention efficacy.

Black plastic ovitraps (diameter 7.5 cm, height 9 cm) with a germination paper (25 cm length, 7 cm height), holding about 150 mL dechlorinated water were placed in release (56) and control areas (20) and in the buffer zone (5) and monitored weekly. Germination papers with eggs were transferred to the laboratory and air dried. After counting eggs, they were allowed to hatch by placing each germination paper into separate bowls with anaerobic water at the insectary. Hatched eggs were counted after 12 h. Adult mosquitoes caught in BG traps placed in the release area (20) and control area (10) were transferred to the laboratory weekly and identified.

### Data management and analysis

Data management in this study was carried out following a rigorous process to ensure accuracy, completeness, and reliability of the dataset. The primary method used was dual-entry, where data were recorded twice by independent operators at different times to minimize transcription errors and increase accuracy. This double-entry method has been widely recognized as an effective approach to reduce data entry errors and ensure the quality of data, particularly in research environments where data integrity is critical [40]. Additionally, custom data entry forms were created using Microsoft Excel 2016, incorporating validation checks at the point of data input. These checks included defining valid input ranges, restricting data types (e.g., numeric or categorical values), and applying validation rules to ensure standardization. Data validation within Excel is a well-established approach that helps ensure consistency, reduce human error, and maintain the quality of collected data [13]. The data were statistically analyzed using R Software, version 3.5.2 (R Development Core Team, Vienna, Austria) [38].

To assess the impact of releasing sterile males on egg density and egg sterility, weekly averages for egg density (eggs per trap) and frequency Ovitrap Index (OI) in both release and control areas were calculated. The OI was determined by dividing the number of ovitraps containing *Aedes* egg or immature mosquitoes by the total number of ovitraps observed. The Ovitrap Density Index (ODI) is the average number of *Aedes* eggs per positive ovitrap [41].

Using a formula for calculating decreased egg Density (D), the relative reduction between the control and release areas was determined as: D = Total eggs per trap per day in the release area - Total eggs per trap per day in the control area / Total eggs per trap per day in the control area × 100.

The percentage of induced egg sterility was estimated using a formula [6] which compares the hatching rates in intervention and control areas. Sterility (S) was calculated as

S =1- (EhI/EI)×(EC/EhC),

where S is the percent egg sterility, Eh represents the mean number of hatched eggs per trap, E is the mean total eggs per trap, I is the release area, and C is the control area.

Furthermore, Poisson-Lognormal Generalized Linear Mixed Models (GLMMs) were utilized. Fixed factors included treatment type, time period, and their interaction, while trap sites and sampling dates were treated as random effects to account for spatial and temporal variations.

Additionally, male mating competitiveness was evaluated using the Fried index, a well-established measure of sterile male efficacy in field conditions. The index was computed using the below equation [15].

Fried index=(W/S)×(PW-PS)/(PS-PRS)

where W and S are the numbers of wild and sterile males, respectively; PW is the percentage of egg hatching in the control area, PS is the percentage in the release area, and PRS is the residual fertility of sterile males, assumed to be 3%.

These metrics make it possible to demonstrate a sustained reduction in egg density and fertility in release areas, indicating the success of sterile male interventions. To further ensure the robustness of results, we applied bootstrapping with 1,000 iterations to estimate the 95% confidence intervals for the Fried index. This method helps assess uncertainty in estimates by resampling the data, and enabling accurate estimation of standard error and confidence intervals [20].

## Results

Field data were collected for 71 weeks (October 2020–August 2022). The results of the field study indicated a 0.82 ± 0.12% female contamination rate of sterile males. This percentage represents the proportion of females released along with sterile males (Table 1).

**Table 1:** Percent of female contamination of released sterile male mosquitoes.

Week	Percent female contamination (%)
01	0.87
02	0.69
03	0.74
04	0.98
05	0.77
06	0.65
07	0.78
08	0.91
09	0.93
10	0.73
11	0.94
12	0.88
13	0.76

### Impact of the IVM strategy on egg density

The impact of the pilot trial on the density of *Ae. albopictus* population was assessed based on the number of eggs collected using ovitraps over 71 weeks. During the 52-week pilot field trial, a total of 8,757 eggs were collected in the control area across all intervention phases, with an average of 6.54 eggs per trap per day with Standard Error (SE) = 0.403. Prior to the intervention, 2,359 eggs were recorded, resulting in a mean of 9.07 eggs per trap per day (SE = 1.26). During intervention part 1, 1,851 eggs were collected, with an average of 7.09 eggs per trap per day (SE = 1.37), followed by a breakdown period with 430 eggs collected, corresponding to an average of 5.31 eggs per trap per day (SE = 1.44). During the intervention part 2, the total reached 2,119 eggs, averaging 6.25 eggs per trap per day (SE = 0.760), and in the post-intervention monitoring phase, 2,998 eggs were collected, with an average of 5.55 eggs per trap per day (SE = 0.510). In the release area, the overall egg collection amounted to 13,397 eggs, with a lower average of 3.72 eggs per trap per day (SE = 0.196), indicating a significant reduction in egg density compared to the control area. During the pre-intervention phase, 3,200 eggs were collected, with an average of 9.12 eggs per trap per day (SE = 1.11). During intervention part 1, 1,961 eggs were recorded, with an average of 5.60 eggs per trap per day (SE = 0.717). The breakdown period yielded 1,375 eggs, with an average of 4.58 eggs per trap per day (SE = 0.828). Intervention part 2 led to the collection of 4,664 eggs, with an average of 3.59 eggs per trap per day (SE = 0.303). Finally, in the post-intervention monitoring phase, 2,197 eggs were collected, with an average of 1.69 eggs per trap per day (SE = 0.175), reflecting a significant population suppression effect in the release area as compared with the control (Table 2).

**Table 2:** Differences in egg density between types of intervention in the control area and release area. Egg density is expressed as a mean number of eggs per trap per day ± SD. (O) Overall eggs collected per trap per day; (A) Pre-intervention; (B) Intervention part 1; (C) Breakdown; (D) Intervention part 2; (E) Post intervention monitoring.

Study area	Variable	O	A	B	C	D	E
Control	Average eggs per trap per day ± SE	6.52±0.40	9.07±1.26	7.09±1.37	5.31±1.44	6.25±0.760	5.55±0.510
Release	Average eggs per trap per day ± SE	3.72±0.196	9.12±1.11	5.60±0.71	4.58±0.828	3.59±0.303	1.69±0.175

### Temporal trends of the Ovitrap Index (OI), Ovitrap Density Index (ODI), and egg density per trap per day

**Figure 2:**
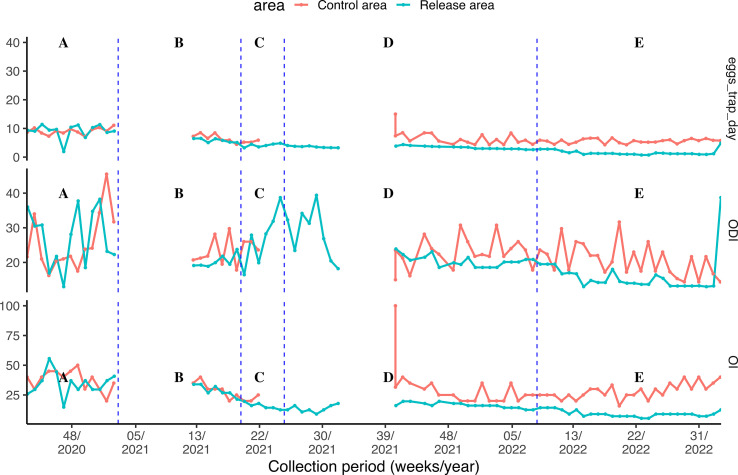
Total number of eggs/trap/day, ODI, and OI by area and collection period. (A) Pre-intervention; (B) Intervention part 1; (C) Breakdown; (D) Intervention part 2; (E) Post-intervention monitoring. ODI – Ovitrap Density Index, OI – Ovitrap Index.

As shown in Figure 2, in the pre-intervention phase, both the control and release areas exhibited fluctuations in mosquito indicators such as eggs per trap per day, the ODI, and the OI. In the control area, egg and density peaks occurred periodically, with notable values in certain weeks, like week 42, where eggs per trap per day reached 8.65, ODI was 21.63, and OI was 40%. The control area’s peak in egg counts and density in week 43 (ODI: 34.0) further indicated higher mosquito densities.

Similarly, the release area showed significant variation, with weeks like week 44 reaching eggs per trap per day values of 11.41 and an ODI of 30.80. The release area also maintained higher OI values compared to the control area, reaching up to 55.56% in week 45, suggesting a higher mosquito prevalence.

### Temporal trend of egg hatch

The temporal trend of egg hatch was observed over 52 weeks in both control and release areas, with an additional focus on the number of eggs collected per trap per day to ensure consistency with part 1.

In the control area, the egg hatch rate ranged from 59.6% to 78.8%, with the highest hatch rate observed during the breakdown period. Across intervention phases, egg density averaged 6.54 eggs per trap per day (SE = 0.403) (Table 3).

**Table 3:** Summary of SIT efficacy during the final 4 weeks of intervention part 2, showing egg hatch rate, eggs per trap per day, and total egg counts in both control and release areas.

	Intervention part 2					
	Control area (20 ovitraps and 10 BG traps)			Release area (56 ovitraps and 20 BG traps)		
Date	Total eggs	Total hatched eggs	Hatch rate	Total eggs	Total hatched eggs	Hatch rate
04/02/2022	87	61	0.70	161	2	1.242
11/02/2022	168	108	0.64	161	2	1.242
18/02/2022	104	79	0.76	146	2	1.37
25/02/2022	118	90	0.76	146	2	1.37
Overall	477	338	0.71	614	8	1.302

**Figure 3:**
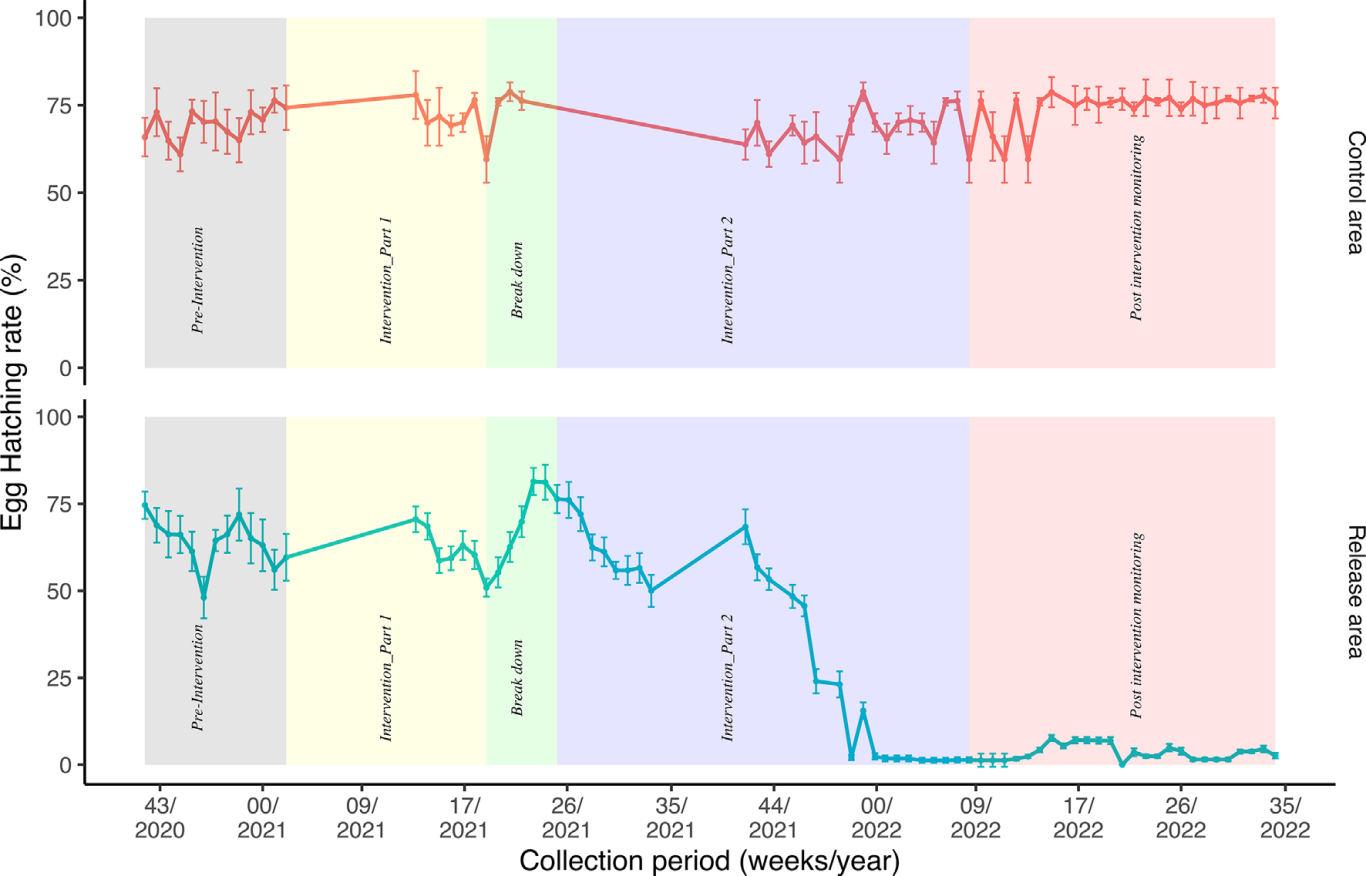
Temporal trend of egg hatching rate in control and release areas.

In the release area, the egg hatch rate ranged from 0% to 81.4%, showing a clear decrease during both intervention phases (intervention parts 1 and 2), with hatch rates dropping close to 0% by the end of intervention part 2. This low rate persisted for 4 consecutive weeks at the start of the post-monitoring phase. However, during the COVID-19 lockdown, when the release of sterile males was paused, the hatch rate began to increase (Fig. 3). The egg density in the release area averaged 3.72 eggs per trap per day (SE = 0.196), reflecting a substantial reduction compared to the control area.

Table 3 summarizes SIT efficacy during the final 4 weeks leading up to the end of intervention part 2, showing egg collection data per trap per day, alongside hatch rates for both control and release areas.

In the post-intervention period, 2,998 eggs were collected in the control area, averaging 5.55 eggs per trap per day (SE = 0.510), with a hatch rate of 74.3%. In the release area, 2,197 eggs were collected, averaging 1.69 eggs per trap per day (SE = 0.175), with a significantly lower hatch rate of 3.23%. Induced egg sterility was calculated at 98.16% in the release area relative to the control area (binomial GLMM, deviance 2.408, df = 2, *p* = 0.016030).

### Temporal trends of adult mosquito abundance (control *vs*. release area)

In the control area, female and male *Ae. albopictus* density did not vary substantially throughout the intervention.

**Figure 4:**
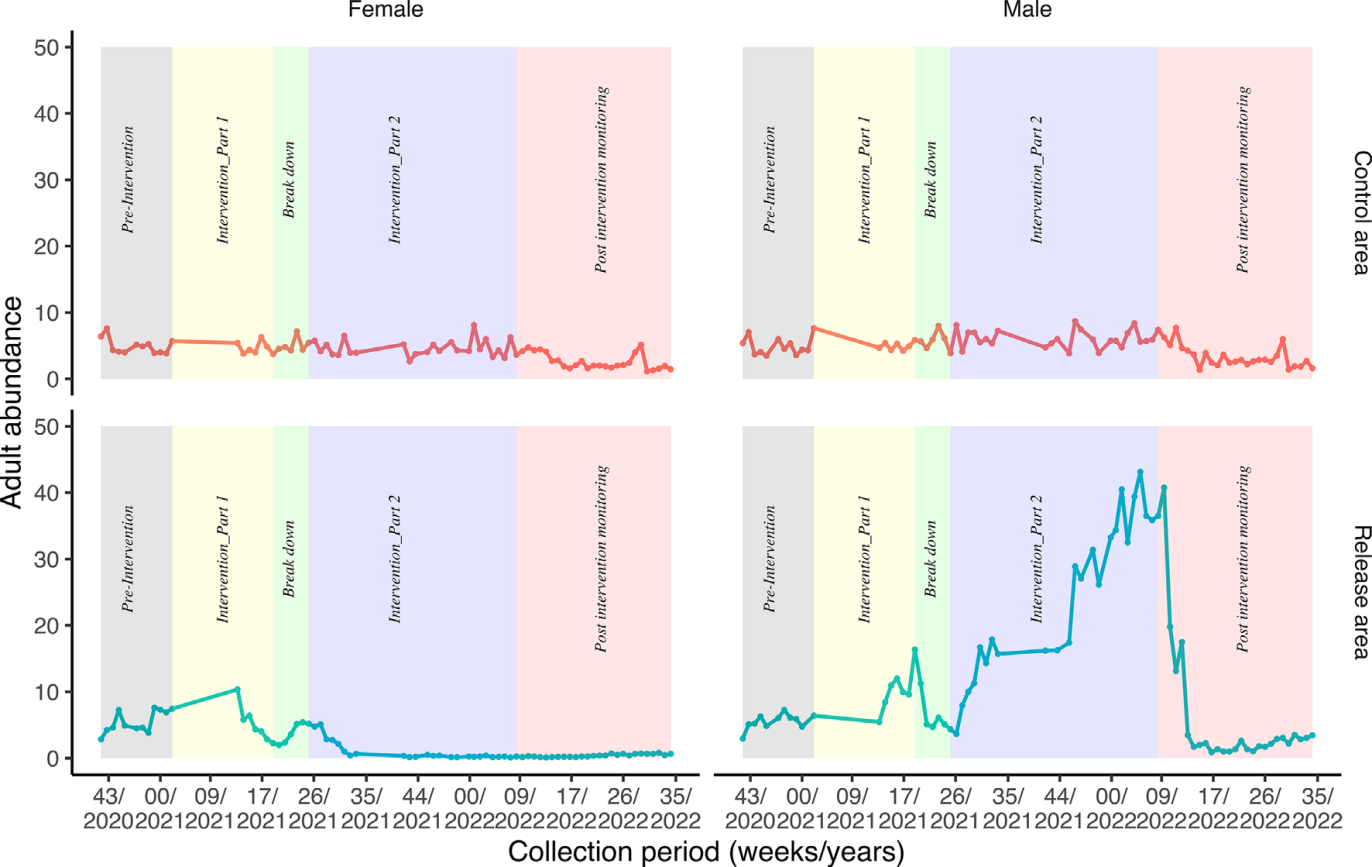
Temporal trend of adult mosquito abundance per trap per week in the control and release areas.

In the intervention area, female density increased up to 10 mosquitoes per trap per week during the first 10 weeks. It started decreasing 6 weeks before intervention part 1, ending with 2 females collected per trap per week. The impact of sterile males started 6 weeks before the end of intervention part 1. During the breakdown period, female density increased due to COVID-19 lockdown, which did not allow continued release of sterile males. The situation was reversed at the beginning of intervention part 2 when the sterile male releases resumed. From week 35/2021 up to week 35/2022, the adult female density was close to zero (Fig. 4). Table 4 shows female adult density reduction 4 consecutive weeks before the end of intervention part 2.

**Table 4:** Female density 4 weeks before the end of intervention part 2.

	Intervention part 2			
	Control area (10 BG traps)	Release area (20 BG traps)	% Suppression (95% CI)	*p*-value
Date	Total females	Total females		
2022-01-28	66	3	95.5 [87.2–98.6]	<0.001**
2022-02-04	86	4	95.3 [88.1–98.3]	<0.001**
2022-02-11	63	5	92.1 [81.3–97.0)]	<0.001**
2022-02-18	126	2	98.4 [94.6–99.6]	<0.001**
Overall	314	14	95.5 [93.2–97.3]	<0.001

** Significant.

**Figure 5:**
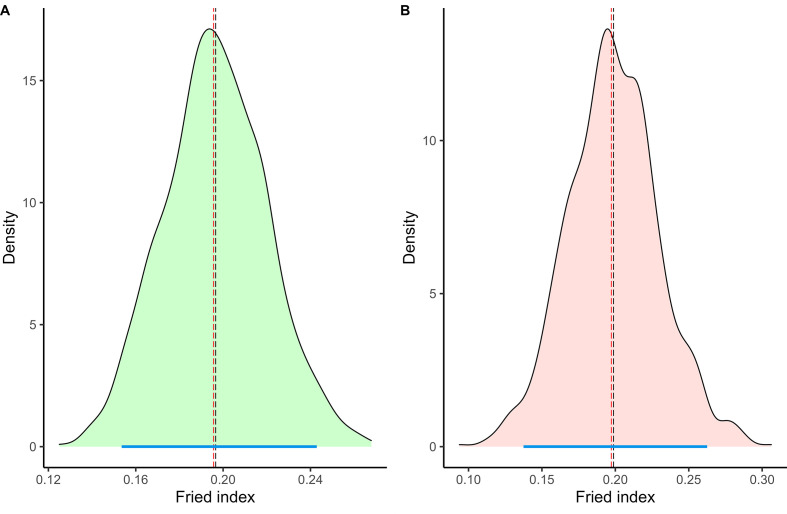
Bootstrap estimate of the Fried index. The long red dash indicates the estimate yield by the observed data, whereas the long black dash shows the bootstrap mean and 95% percentile interval in the blue line. (A) and (B) are intervention part 1 and 2 estimation of the Fried index from 1,000 bootstraps in the distributions of sterile-to-wild male ratios in the control and release areas, respectively. The density corresponds to the percentage of the simulations for a given value.

To quantify sterile male release efficacy, the density reduction was computed between release and control areas during 4 consecutive weeks and 95.5% suppression (95% CI: 93.2–97.3%; *p* < 0.001) was observed in the release area. The suppression remained for 6 months after stopping the releases (Fig. 5).

### Competitiveness index

**Figure 6:**
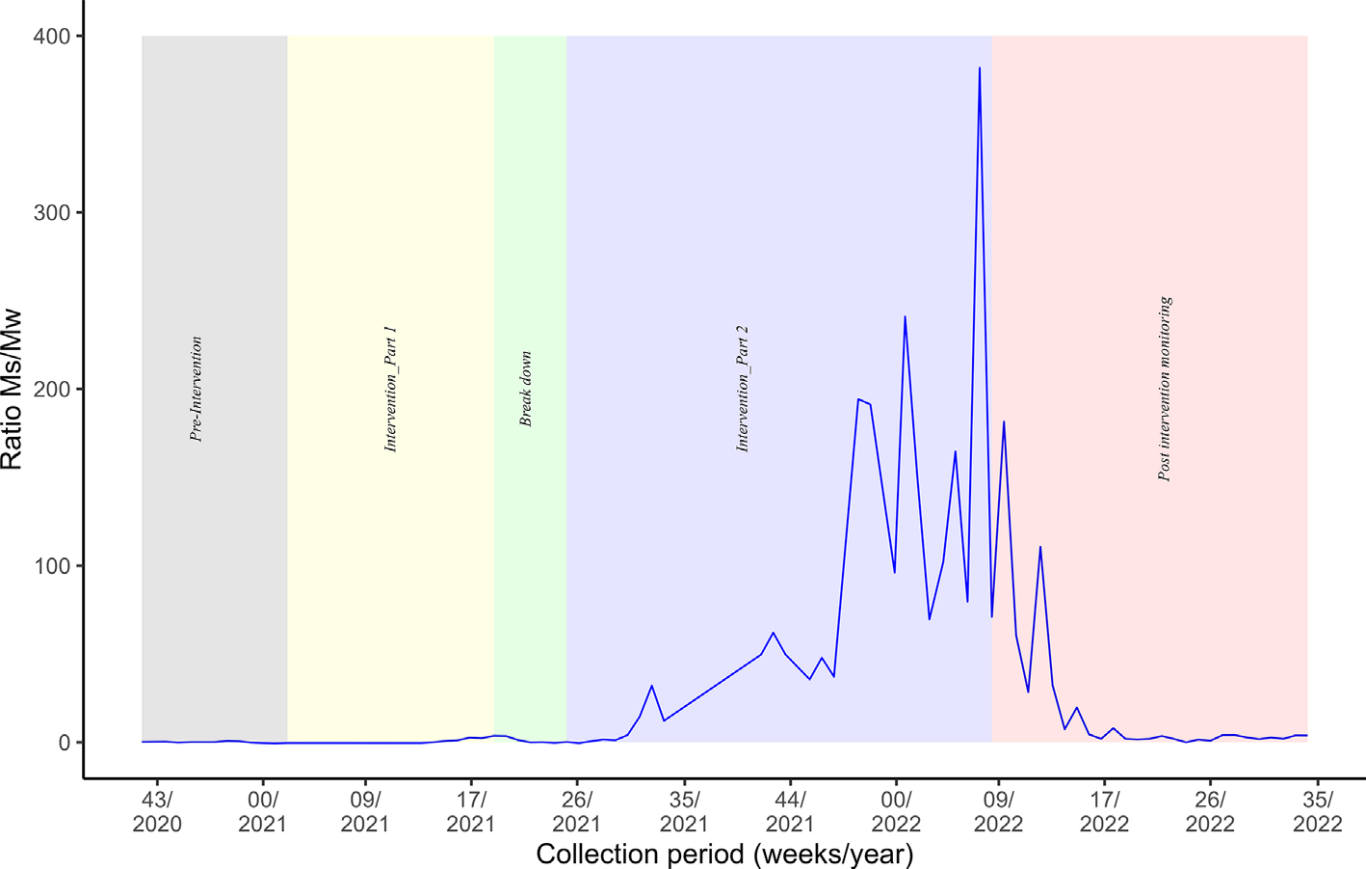
Temporal trend of the ratio of sterile to wild males.

During intervention part 1, the sterile-to-wild male ratio was consistently less than 4, allowing a good evaluation of the competitiveness index. The estimated Wild/Sterile (W/S) ratio was calculated to be 1.1300 ± 0.8263, PW was 0.726698; PS was 0.620932, and PRS was 0.01. The Fried index for this period was evaluated at 0.19 (95% confidence interval 0.15, 0.24) (Fig. 6).

In intervention part 2, the S/W male ratio was below 4 only during weeks 26, 27, 28, and 29, allowing an estimation of the Fried index. The estimated W/S ratio during this time was 1.9055 ± 0.1988, with a PW of 0.7540, PS of 0.6842, and PRS of 0.01. The Fried index was assessed at 0.19 (95% confidence interval: 0.13–0.26).

For the remainder of intervention part 2, the sterile-to-wild male ratio exceeded the threshold necessary for estimating the Fried index at 27. Specifically, this ratio exceeded a value of 10 after week 30, reaching 100 after week 32, and surpassing 300 by the end of the intervention period. Throughout this phase, fluctuations were observed (Fig. 1).

## Discussion

A case control study was conducted under the current pilot field trial (Phase II) in the Gampaha district in Sri Lanka where the second highest number of dengue cases was reported.

The current study demonstrated successful application of SIT for the suppression of *Ae. albopictus*. Similarly, the SIT has been utilized successfully to suppress *Ae. albopictus* dengue vector mosquitoes in different parts of the world: Italy [6, 7], Reunion Island [19, 36], Mauritius [25], Greece [3, 4], Germany [5], and Spain [44] as well as *Ae. aegypti* in Cuba [16, 17].

The selection of an appropriate pilot study site is critical for obtaining solid data [25, 37]. Both release and control areas need to be selected based on easy logistic management, geographical isolation, and presence of only the target *Aedes* species. In the current study, geographically isolated areas of 30 ha were selected, with similar size, number of households, and socio-economic conditions together with the presence of only *Ae. albopictus* based on previous entomological surveillance data.

The trial was disturbed due to the COVID-19 lockdown. Therefore, the first intervention (part 1) was carried out for 8 weeks and resulted in about 30% reduction in egg fertility. Intervention part 2 was carried out for 18 weeks and resulted in a 98% reduction in egg fertility compared to the control (Fig. 3). The suppression remained for 6 months after stopping the releases. These results showed a clear impact of the release of sterile males in the release area. Adult mosquito abundance showed a reduction of 95.54% in the release area as compared with the control area. SIT was the only intervention carried out during the study period. Both intervention and control areas were situated close to each other; therefore, both areas had similar meteorological conditions. Pilot field trials conducted for *Ae. albopictus* in other countries reported high reduction in egg fertility, 70–80% in Italy [6], 84.7% in Germany [5], and 78% in Spain [44].

The Fried index in the field should at least 0.2 to have good efficiency [8]. In our study, the Fried index for the two intervention periods were actually close to 0.2. The current study showed good suppression during the pilot trial in Sri Lanka, even though the Fried index was only 0.2.

During intervention part 1, a general decrease in eggs per trap per day was noted in the control area, particularly between weeks 15 and 18, while OI values stabilized, suggesting the intervention’s potential in lowering the mosquito population. In the release area, eggs per trap per day values remained lower, such as 5.05 in week 15, with moderate OI values. During the breakdown phase, the control area showed a marked decrease in some weeks, such as week 19, with eggs per trap per day dropping to 4.45. However, a spike occurred in week 41, with eggs per trap per day reaching 15 and OI 100%, indicating a temporary increase in mosquito presence despite prior intervention efforts. The release area exhibited lower values in weeks 20–24, exemplified by an eggs per trap per day of 3.23 in week 20, but also experienced an increase in week 25, with ODI reaching 38.71, suggesting a slight resurgence in mosquito density. During intervention part 2, the control area displayed egg densities ranging between 4.35 and 8.4 from weeks 41 to 52. In the release area, egg densities remained consistently low, typically below 5, with ODI values ranging from 20 to 40. This may be due to seasonal trends in mosquito density. During post-intervention monitoring, eggs per trap per day in the release area stabilized and remained reduced over time, with both ODI and OI showing sustained changes across both areas. The control area’s metrics, however, offer insight into environmental or seasonal variations, which might influence mosquito density trends independently of the intervention.

Impact of SIT on egg density showed the release area had a 69% lower egg density as compared to the control area. Moreover, using a Poisson-log normal GLMM to account for variability and overdispersion, the adjusted reduction in egg density between the areas was estimated to be 58.80% (*p* = 0.000004).

The adjusted reduction in egg density, coupled with the observed induced sterility of 98.16%, led to a female adult density reduction of 95.54% in the release area for 4 consecutive weeks before intervention part 2 ended. Releasing 100,000 sterile males per week could have induced a greater and quicker suppression of the local mosquito population if the competitiveness index was higher than observed in this study. An effort to improve quality is thus required, based on the review of the handling/irradiation/release procedures. Nevertheless, release numbers allowed more than 80% sterility in the wild *Ae. albopictus* population, which prevents the occurrence of compensation and over-compensation in larval mortality [10]. The very high male to female ratios (>300) at the end of the intervention period may have contributed to reduce the female density through mating harassment, as recently demonstrated in China [49].

In this study, there was a 0.82 ± 0.12% female contamination rate of sterile males. The presence of released females can undermine the effectiveness of SIT by contributing to the local pest population, as these females can mate with fertile males. Residual female presence of 0.71 ± 0.35% was reported in Greece [32] and 0.17% in Spain [44].

This contamination can occur due to errors in the sex-sorting process or inadequate sterilization procedures. Minimizing female contamination is crucial for the success of SIT programs, as it ensures that the released sterile males have the maximum impact on reducing pest populations. Using automated sorters is crucial to upscale SIT against mosquitoes [18, 33].

This pilot field trial was conducted in a semi-urban area. There is a scalability to conduct a pre-operational trial with entomological and epidemiological monitoring covering a larger area (600 ha) will be initiated to suppress wild populations and potentially reduce disease transmission.

In conclusion, the pilot field trial (Phase II) with *Ae. albopictus* resulted in strong suppression of vector mosquitoes, which persisted for 6 months after stopping the releases. This is an unprecedented result that we attribute to the strong isolation of the release area, surrounded by rice crops (Fig. 1) and one wide road. Results of the pilot field trial allowed us to fulfil all milestones of the PCA for mosquito SIT [9, 45]. Based on the successful results of this trial, an SIT pre-operational trial with entomological and epidemiological monitoring covering a larger area will be initiated.

In case of success, as recently observed in Singapore [31], SIT will be integrated into future IVM programs in the country as an additional powerful tool to control *Aedes* vectors together with source reduction, community awareness, and other vector control methods.

## Data Availability

Some data are provided within the manuscript or supplementary information files; others are on file and stored by the corresponding author.

## Supplementary material

*File S1*. Location of BG and ovitraps. *File S2*. Adult density and calculations in the release area. *File S3*. Adult density and calculations in the control area. The supplementary material for this article can be found at https://www.parasite-journal.org/10.1051/parasite/2025050/olm.
