# Supplementary material for: Suppression of Aedes albopictus in Sri Lanka using the Sterile Insect Technique (SIT) with a sustained effect
Source: Parasite. 2025 Sep 17;32:59. doi: 10.1051/parasite/2025050 (PMC12443424; doi:10.1051/parasite/2025050)
Supplement: Supplementary file 1 — File S1. Location of BG and ovitraps. File S2. Adult density and calculations in the release area. File S3. Adult density and calculations in the control area. [file parasite-32-59-s1.pdf]

Trap type	Area	Name	Latitude	Longitude
Ovitrap	release area	T1	7.082003	80.010900
Ovitrap	release area	T2	7.081397	80.011258
Ovitrap	release area	T3	7.080933	80.011497
Ovitrap	release area	T4	7.080242	80.011792
Ovitrap	release area	T5	7.078900	80.012331
Ovitrap	release area	T6	7.078378	80.012731
Ovitrap	release area	T7	7.078333	80.012014
Ovitrap	release area	T8	7.078950	80.011678
Ovitrap	release area	T9	7.079547	80.011386
Ovitrap	release area	T10	7.079758	80.011936
Ovitrap	release area	T11	7.080014	80.010783
Ovitrap	release area	T12	7.080114	80.011197
Ovitrap	release area	T13	7.080283	80.010425
Ovitrap	release area	T14	7.079867	80.010494
Ovitrap	release area	T15	7.080311	80.010003
Ovitrap	release area	T16	7.080453	80.009450
Ovitrap	release area	T17	7.080686	80.009844
Ovitrap	release area	T18	7.080911	80.009333
Ovitrap	release area	T19	7.081050	80.009728
Ovitrap	release area	T20	7.080847	80.009025
Ovitrap	release area	T21	7.081219	80.008828
Ovitrap	release area	T22	7.081256	80.008197
Ovitrap	release area	T23	7.081097	80.007686
Ovitrap	release area	T24	7.080992	80.006858
Ovitrap	release area	T25	7.080475	80.006528
Ovitrap	release area	T26	7.080672	80.005889
Ovitrap	release area	T27	7.079750	80.006375
Ovitrap	release area	T28	7.079250	80.006811
Ovitrap	release area	T29	7.079014	80.007303
Ovitrap	release area	T30	7.079133	80.008308
Ovitrap	release area	T31	7.080014	80.007753
Ovitrap	release area	T32	7.080131	80.008694
Ovitrap	release area	T33	7.078139	80.008453
Ovitrap	release area	T34	7.077525	80.008761
Ovitrap	release area	T35	7.077806	80.009408
Ovitrap	release area	T36	7.078228	80.009908
Ovitrap	release area	T37	7.078942	80.009831
Ovitrap	release area	T38	7.078494	80.010492
Ovitrap	release area	T39	7.079372	80.009200
Ovitrap	release area	T40	7.078439	80.009133
Ovitrap	release area	T41	7.079208	80.010719
Ovitrap	release area	T42	7.079292	80.010211
Ovitrap	release area	T43	7.078689	80.011339

Ovitrap	release area	T44	7.078039	80.011753
Ovitrap	release area	T45	7.078856	80.010131
Ovitrap	release area	T46	7.080789	80.010817
Ovitrap	release area	T47	7.080906	80.010328
Ovitrap	release area	T48	7.081411	80.010550
Ovitrap	release area	T49	7.082003	80.010267
Ovitrap	release area	T50	7.081667	80.010108
Ovitrap	release area	T51	7.081586	80.009628
Ovitrap	release area	T52	7.081617	80.009186
Ovitrap	release area	T53	7.080358	80.008281
Ovitrap	release area	T54	7.079653	80.007733
Ovitrap	release area	T55	7.079847	80.006944
Ovitrap	control area	C1	7.080355	80.018941
Ovitrap	control area	C2	7.081142	80.018915
Ovitrap	control area	C3	7.082254	80.019686
Ovitrap	control area	C4	7.083021	80.019841
Ovitrap	control area	C5	7.079374	80.018062
Ovitrap	control area	C6	7.080446	80.017978
Ovitrap	control area	C7	7.079340	80.018909
Ovitrap	control area	C8	7.079457	80.019878
Ovitrap	control area	C9	7.079419	80.020614
Ovitrap	control area	C10	7.081125	80.019882
Ovitrap	control area	C11	7.082013	80.020723
Ovitrap	control area	C12	7.082104	80.021682
Ovitrap	control area	C13	7.081266	80.021515
Ovitrap	control area	C14	7.081196	80.020751
Ovitrap	control area	C15	7.080306	80.021561
Ovitrap	control area	C16	7.079472	80.021500
Ovitrap	control area	C17	7.078590	80.019869
Ovitrap	control area	C18	7.078546	80.018948
Ovitrap	control area	C19	7.080403	80.020803
Ovitrap	control area	C20	7.080318	80.019808
BG trap	release area	BGS1	7.080227	80.006207
BG trap	release area	BGS2	7.079447	80.006697
BG trap	release area	BGS3	7.079304	80.008105
BG trap	release area	BGS4	7.077507	80.009206
BG trap	release area	BGS5	7.078523	80.009640
BG trap	release area	BGS6	7.079574	80.010020
BG trap	release area	BGS7	7.079501	80.011009
BG trap	release area	BGS8	7.079551	80.009395
BG trap	release area	BGS9	7.080092	80.008374
BG trap	release area	BGS10	7.080859	80.007183
BG trap	release area	BGS11	7.078195	80.011906
BG trap	release area	BGS12	7.081325	80.009640

BG trap	release area	BGS13	7.078962	80.012221
BG trap	release area	BGS14	7.079806	80.011896
BG trap	release area	BGS15	7.081043	80.011307
BG trap	release area	BGS16	7.081891	80.010886
BG trap	release area	BGS17	7.081197	80.010381
BG trap	release area	BGS18	7.081425	80.008591
BG trap	release area	BGS19	7.080355	80.009608
BG trap	release area	BGS20	7.080000	80.006944
BG trap	control area	BGC1	7.080833	80.018611
BG trap	control area	BGC2	7.081667	80.020000
BG trap	control area	BGC3	7.079167	80.020000
BG trap	control area	BGC4	7.080833	80.020833
BG trap	control area	BGC5	7.079167	80.021389
BG trap	control area	BGC6	7.080278	80.021944
BG trap	control area	BGC7	7.082305	80.020898
BG trap	control area	BGC8	7.079167	80.018889
BG trap	control area	BGC9	7.080556	80.019722
BG trap	control area	BGC10	7.078611	80.019444
